# The activation of G protein-coupled receptor 30 (GPR30) inhibits proliferation of estrogen receptor-negative breast cancer cells *in vitro* and *in vivo*

**DOI:** 10.1038/cddis.2014.398

**Published:** 2014-10-02

**Authors:** W Wei, Z-J Chen, K-S Zhang, X-L Yang, Y-M Wu, X-H Chen, H-B Huang, H-L Liu, S-H Cai, J Du, H-S Wang

**Affiliations:** 1 Guangdong Provincial Key Laboratory of New Drug Design and Evaluation, Department of Microbial and Biochemical Pharmacy, School of Pharmaceutical Sciences, Sun Yat-sen University, Guangzhou 510006, China; 2 Department of Pharmacy, Sun Yat-sen University Cancer Center; State Key Laboratory of Oncology in South China; Collaborative Innovation Center for Cancer Medicine, Guangzhou 510060, China; 3 Department of Pharmacy, Sun Yat-sen Memorial Hospital, Sun Yat-sen University, 107 Yanjiang West Road, Guangzhou 510120, China; 4 Key Laboratory of Tropical Disease Control (Ministry of Education), Guangdong Institute of Gastroenterology and the Sixth Affiliated Hospital, Institute of Human Virology, Sun Yat-sen University, Guangzhou 510655, China; 5 Department of Pharmacology, School of Pharmaceutical Sciences, Jinan University, Guangzhou 510632, China

## Abstract

There is an urgent clinical need for safe and effective treatment agents and therapy targets for estrogen receptor negative (ER−) breast cancer. G protein-coupled receptor 30 (GPR30), which mediates non-genomic signaling of estrogen to regulate cell growth, is highly expressed in ER− breast cancer cells. We here showed that activation of GPR30 by the receptor-specific agonist G-1 inhibited the growth of ER− breast cancer cells *in vitro*. Treatment of ER− breast cancer cells with G-1 resulted in G2/M-phase arrest, downregulation of G2-checkpoint regulator cyclin B, and induction of mitochondrial-related apoptosis. The G-1 treatment increased expression of p53 and its phosphorylation levels at Serine 15, promoted its nuclear translocation, and inhibited its ubiquitylation, which mediated the growth arrest effects on cell proliferation. Further, the G-1 induced sustained activation and nuclear translocation of ERK1/2, which was mediated by GPR30/epidermal growth factor receptor (EGFR) signals, also mediated its inhibition effects of G-1. With extensive use of siRNA-knockdown experiments and inhibitors, we found that upregulation of p21 by the cross-talk of GPR30/EGFR and p53 was also involved in G-1-induced cell growth arrest. *In vivo* experiments showed that G-1 treatment significantly suppressed the growth of SkBr3 xenograft tumors and increased the survival rate, associated with proliferation suppression and upregulation of p53, p21 while downregulation of cyclin B. The discovery of multiple signal pathways mediated the suppression effects of G-1 makes it a promising candidate drug and lays the foundation for future development of GPR30-based therapies for ER− breast cancer treatment.

Breast cancer is the most frequently diagnosed cancer and the leading cause of cancer death in females worldwide.^1^ Clinically, breast cancer is generally classified into estrogen receptor *α* positive (ER+) or ER-negative (ER−) subtypes.^2^ ER− tumors are often intrinsically more aggressive and of higher grade than ER+ tumors.^3^ Since lack of the effectiveness of ER-targeted endocrine treatments (tamoxifen and aromatase inhibitors), patients with ER− breast cancer have significantly worse prognosis and greater 5-year recurrence rate than that of ER+ breast cancer.^4^ Considering that ER− breast cancer constitutes around 30% of all breast cancers,^5^ there is an urgent need to explore new targeted approaches for its treatment.

A seven-transmembrane receptor G protein-coupled receptor 30 (GPR30), which is structurally unrelated to nuclear ER, has been recently shown to mediate rapid non-genomic signals of estrogens. The activation of GPR30 can stimulate adenylyl cyclase, transactivate epidermal growth factor receptors (EGFRs), induce mobilization of intracellular calcium (Ca^2+^) stores, and activate mitogen-activated protein kinase (MAPK) and phosphoinositide 3-kinase (PI3K) signaling pathways.^6,7^ Previous studies revealed that GPR30 can modulate growth of hormonally responsive cancers such as endometrial,^8^ ovarian,^9^ and breast cancer.^10^ Therefore, GPR30 likely has an important role in modulating estrogen responsiveness and development and/or progression of ER− breast cancer. Studies revealed that activation of GPR30 can induce the expression of genes and activate pathways that facilitate cell proliferation of endometrial,^11,12^ breast,^13^ and ovarian cancer.^14^ On the contrary, numerous studies demonstrated that activation of GPR30 by its specific agonist G-1 results in cell-cycle arrest and proliferation inhibition of ER*α*-positive breast cancer,^10^ endothelial cells,^15^ prostate,^16^ and ovarian^9^ cancer cells. So it requires further investigation on the function of activating GPR30 and the effect of G-1 on the cancer cells.

GPR30 has been reported to be expressed in ER− breast cancer cells and suggested to be an excellent new therapeutic target for the treatment of ER− breast cancer.^17^ Confusedly, the only two published papers reported contradictory results: Girgert *et al.*^18^ stated that activation of GPR30 promotes growth of ER− breast cancer cells, while Weissenborn *et al.*^19^ revealed that GPR30 functions as a tumor suppressor of ER− breast cancer cells. Therefore, there is an urgent need to illustrate the effects of GPR30 on the proliferation of ER− breast cancer and its downstream signal mechanisms. In the present study, we demonstrated that activation of GPR30 by G-1 inhibits the proliferation of ER− breast cancer cells both *in vitro* and *in vivo*.

## Results

### Activation of GPR30 inhibited ER− breast cancer cell growth *in vitro*

**Figure 1:**
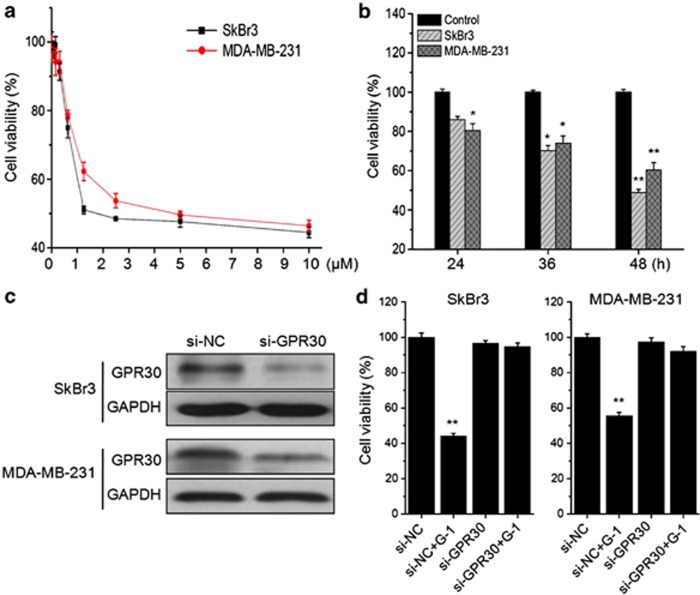
The activation of GPR30 inhibited the proliferation of ER− breast cancer cells. (**a**) SkBr3 and MDA-MB-231 cells were treated with various concentrations (10^−8^ to 10^−5^ M) of G-1 for 48 h, and then cell viability was assessed by CCK-8 kit. (**b**) SkBr3 and MDA-MB-231 cells were treated with 1 *μ*M G-1 for 24, 36, and 48 h, respectively, and then cell viability was assessed by CCK-8 kit. (**c**) After 24 h pre-transfection with si-NC or si-GPR30 siRNAs, the protein levels of GPR30 were analyzed by western blotting. (**d**) After 24 h pre-transfection with si-NC or si-GPR30 siRNAs, SkBr3 or MDA-MB-231 cells were further treated with 1 *μ*M G-1 for 48 h, and then cell viability was assessed by CCK-8 kit. Data were presented as means±S.D. of three independent experiments (ten independent experiments for cell viability). *P<0.05 compared with control; **P<0.01 compared with control

Two ER− breast cancer cell lines, SkBr3 (ER*α*− and ER*β*−) and MDA-MB-231 (ER*α*− and ER *β*+), were treated with G-1 to study the activation of GPR30 on cell proliferation. We found that activation of GPR30 by G-1 for 48 h significantly inhibited the proliferation of both SkBr3 and MDA-MB-231 cells *via* a concentration-dependent manner (Figure 1a ). The IC_50_ values of G-1 (48 h) to SkBr3 and MDA-MB-231 cells were 3.69 and 5.13 *μ*M, respectively. Therefore, 1 *μ*M G-1 was chose for further studies on the basis of cytotoxicity test and other previous studies.^13,20^ We found that G-1 also inhibited growth of both MDA-MB-231 and SkBr3 *via* a time-dependent manner (Figure 1b). Then, we performed knockdown GPR30 assay in both SkBr3 and MDA-MB-231 cells (Figure 1c). The silence of GPR30 significantly attenuated G-1 induced proliferation suppression for both SkBr3 and MDA-MB-231 cells (Figure 1d). Collectively, these data revealed that activation of GPR30 by agonist G-1 can significantly inhibit the *in vitro* growth of ER− breast cancer cells.

### Activation of GPR30 induced G2/M cell-cycle arrest

**Figure 2:**
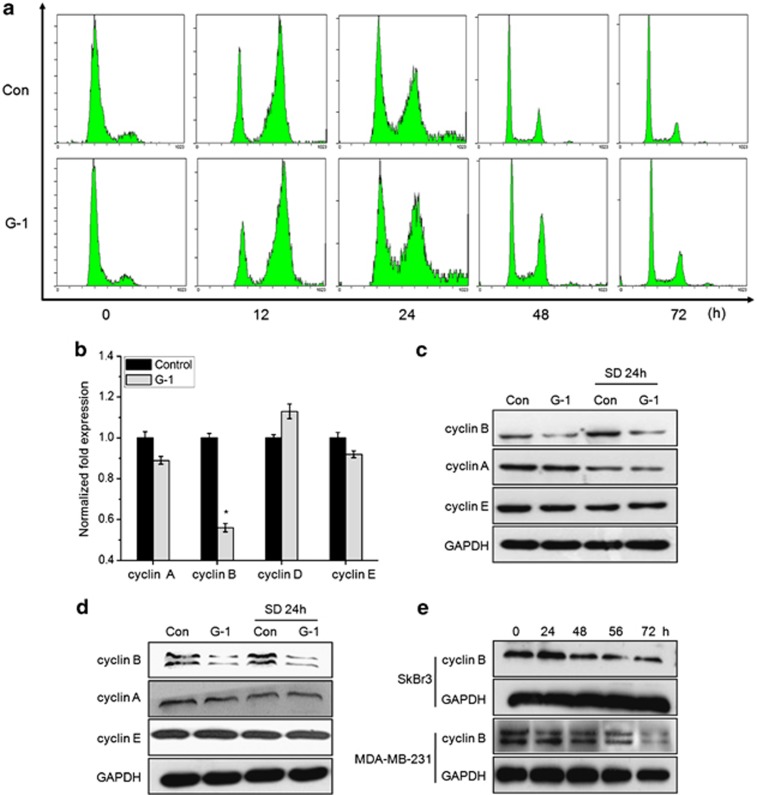
The activation of GPR30 induced G2/M cell-cycle arrest. (**a**) SkBr3 cells were synchronized at the G1/S transition by a double TdR block, and then treated with 1 *μ*M G-1 for the indicated times. The cycle cycles were analyzed by FCM; (**b**) SkBr3 cells were treated with 1 *μ*M G-1 for 24 h, and then the mRNA levels of cyclins were measured by qRT-PCR; SkBr3 (**c**) and MDA-MB-231 (**d**) cells were treated with 1 *μ*M G-1 for 72 h, and then protein levels of cyclins were analyzed by western blotting; (**e**) SkBr3 and MDA-MB-231 cells were treated with 1 *μ*M G-1 for the indicated times, the protein levels of cyclin B were measured by western blotting. Data were presented as means±S.D. of three independent experiments. *P<0.05 compared with control

Whether activation of GPR30 blocked cells in a specific phase of cell cycle was further determined. We synchronized cells using double TdR-blocking method so that cells can come in a same stage. Flow-cytometric analysis showed a significant (*P*<0.05) increase in the number of cells in G2/M phase after treatment of SkBr-3 cells with G-1 for 12 h. The increase in G2/M phases by G-1 lasted throughout 72-h treatment period (Figure 2a). Similar G2/M arrest by G-1 was also observed in MDA-MB-231 cells (data not shown). Further, we found that instead of cyclin A, cyclin D, and cyclin E, G-1 treatment significantly (*P*<0.05) decreased the mRNA of cyclin B in SkBr3 cells (Figure 2b). It was confirmed by western blotting that G-1 treatment resulted in a significant (*P*<0.05) reduction in the protein levels of cyclin B in both SkBr3 (Figure 2c) and MDA-MB-231 (Figure 2d) cells. This reduction was more stronger in cells serum deprived for 24 h before G-1 treatment. Further, G-1 can inhibit protein levels of cyclin B in a time-dependent manner in both SkBr3 and MDA-MB-231 cells (Figure 2e). Consequently, downregulation of cyclin B gave a direct insight into G-1 induce G2/M cell-cycle arrest *via* impair the G2/M transition.

### Activation of GPR30 induced mitochondrial-related apoptosis

**Figure 3:**
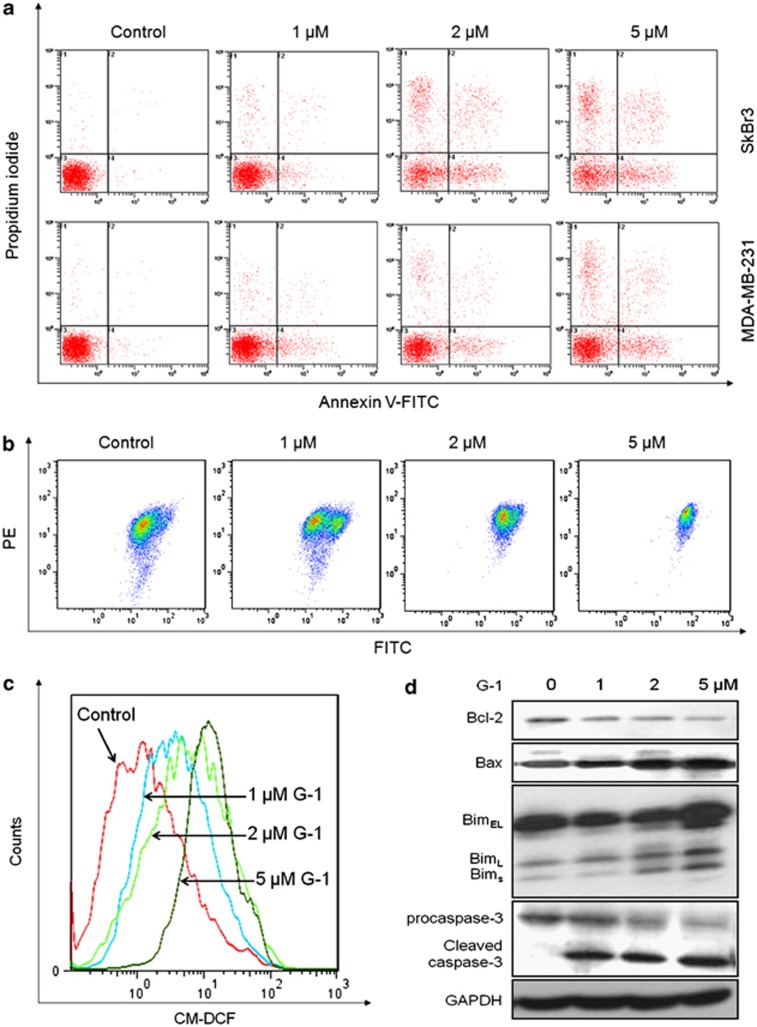
The activation of GPR30 induced mitochondrial-related apoptosis. (**a**) SkBr3 and MDA-MB-231 cells were treated with increasing concentrations of G-1 for 48 h, stained with annexin V-FITC and PI, and then analyzed by flow cytometry for cell apoptosis. (**b**) SkBr3 cells were treated with G-1 as the indicated concentrations for 24 h, and then JC-1, the mitochondria-specific dye, was added to measure the membrane polarity (ΔΨm) and cell apoptosis. Apoptotic cells mainly show green fluorescence (FITC), while healthy cells show red fluorescence (PE). (**c**) SkBr3 cells were treated with various concentrations of G-1 for 4 h, and then loaded with CM-H_2_DCFDA. The fluorescence intensity was measured by FCM. (**d**) SkBr3 cells were treated with G-1 as the indicated concentrations for 48 h, and then Bcl-2, Bax, Bim, and caspase-3 protein expression levels were analyzed by western blotting. Data were presented as means±S.D. of three independent experiments

As shown in Figure 3a, G-1 treatment resulted in a marked dose-dependent increase in apoptosis of both SkBr3 and MDA-MB-231 cells. Our results showed that G-1 treatment deceased the mitochondrial membrane potential (ΔΨm) *via* a concentration-dependent manner (Figure 3b). In addition, treatment with G-1 significantly increased the reactive oxygen species (ROS) generation in a dose-dependent manner (Figure 3c). The apoptotic-related proteins were further measured. As shown in Figure 3d, activation of GPR30 significantly (*P*<0.05) upregulated the expression of Bax, Bim, and cleaved caspase-3, while downregulated the expression of Bcl-2 and procaspase-3. Collectively, these data suggested that the mitochondrial-related apoptosis was involved in G-1-induced ER− breast cancer cell growth arrest.

### p53 mediated growth arrest of G-1 in ER− breast cancer cells

**Figure 4:**
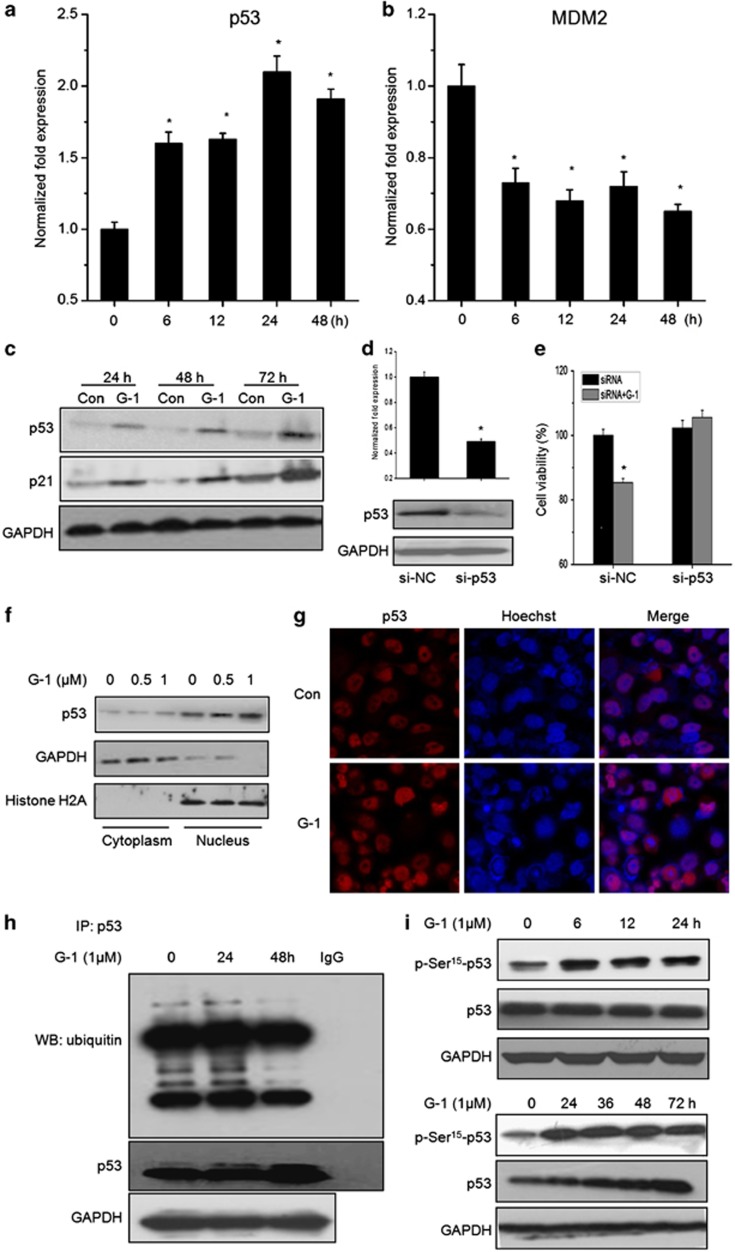
p53 mediated growth arrest of G-1 in ER− breast cancer cells. SkBr3 cells were treated with 1 *μ*M G-1 for the indicated time periods, and then mRNA levels of p53 (**a**) and MDM2 (**b**) were quantified by real-time PCR, the protein levels of p53 (**c**) were detected by western blotting. SkBr3 cells transfected with si-p53 or si-NC for 24 h, and the mRNA and protein expression of p-53 were measured by qRT-PCR and western blotting (**d**), respectively. The transfected cells were then stimulated with or without G-1 (1 *μ*M) for another 24 h, the cell viability was assessed by CCK-8 kit (**e**). (**f**) After treatment with G-1 for 24 h, nuclear and cytoplasmic cellular fractions were isolated by differential lysis. The levels of p53 in nuclear and cytoplasmic cellular fractions were detected by western blotting. (**g**) SkBr3 cells were treated with or without G-1 (1 *μ*M) for 24 h. After fixation, the cellular location of p53 (red) was examined by immunofluorescence staining and nuclei were stained with Hoechst (blue). (**h**) SkBr3 cells treated with 1 *μ*M G-1 for the indicated time periods. After p53 was immunoprecipitated from equal amount of lysates, the ubiquitination of p53 was examined by Western blotting. (**i**) SkBr3 cells were treated with 1 *μ*M G-1 for the indicated time periods, and then p-Ser^15^-p53 and p53 were measured by western blotting. Data were presented as means±S.D. of three independent experiments. *P<0.05 compared with control

The p53-mediated pathway is one of the key regulatory pathways of ER− breast cancer development.^21^ Our results revealed that G-1 treatment significantly increased the mRNA expression of p53 (Figure 4a). The mRNA levels of MDM2, which can promote the rapid degradation of p53,^22^ were significantly decreased by G-1 treatment (Figure 4b) *via* a time-dependent manner. Also, G-1 increased the protein expression of both p53 and p21 in SkBr3 cells (Figure 4c). Knockdown assays were performed to verify that p53 is a key regulator in G-1-induced growth arrest of ER− breast cancer cells. Both mRNA and protein levels of p53 were successfully silenced by si-p53 (Figure 4d). As shown in Figure 4e, silencing of p53 significantly attenuated G-1-induced growth arrest of SkBr3 cells, which was not observed in control siRNA-transfected cells. The results revealed that p53 mediated the growth arrest of G-1 in ER− breast cancer cells.

The cellular location and post-translational modification of p53 stimulated by G-1 were further investigated to study the p53-dependent mechanisms. Our results revealed that G-1 treatment increased the abundance of p53 in the cell nucleus (Figure 4f). The results of immunofluorescence confirmed that activation of GPR30 by G-1 significantly increased the nuclear translocation of p53 (Figure 4g). Because protein stability of p53 is regulated *via* ubiquitin mediated proteasomal degradation processes,^23^ ubiquitination state of p53 was detected by western blotting with an anti-ubiquitin antibody. The results revealed that activation of GPR30 by G-1 dramatically suppressed the ubiquitylation of p53 *via* a time-dependent manner (Figure 4h). It was reported that p53 phosphorylated by AMPK on Ser 15 (p-Ser^15^-p53) is essential for its translocation to the nucleus.^24^ Our results showed that G-1 significantly (*P*<0.05) increased the levels of p-Ser^15^-p53 since 6 h. The upregulation of p-Ser^15^-p53 by G-1 lasted for more than 72 h. Further, G-1 treatment upregulated total p53 *via* a time-dependent manner (Figure 4i). Collectively, our results suggested that activation of GPR30 by G-1 increased mRNA, protein, and phosphorylation levels of p53, promoted its nuclear translocation, inhibited its ubiquitylation, and then suppressed the growth of ER− breast cancer cells.

### Activation of ERK by GPR30/EGFR mediated the growth arrest effects of G-1

**Figure 5:**
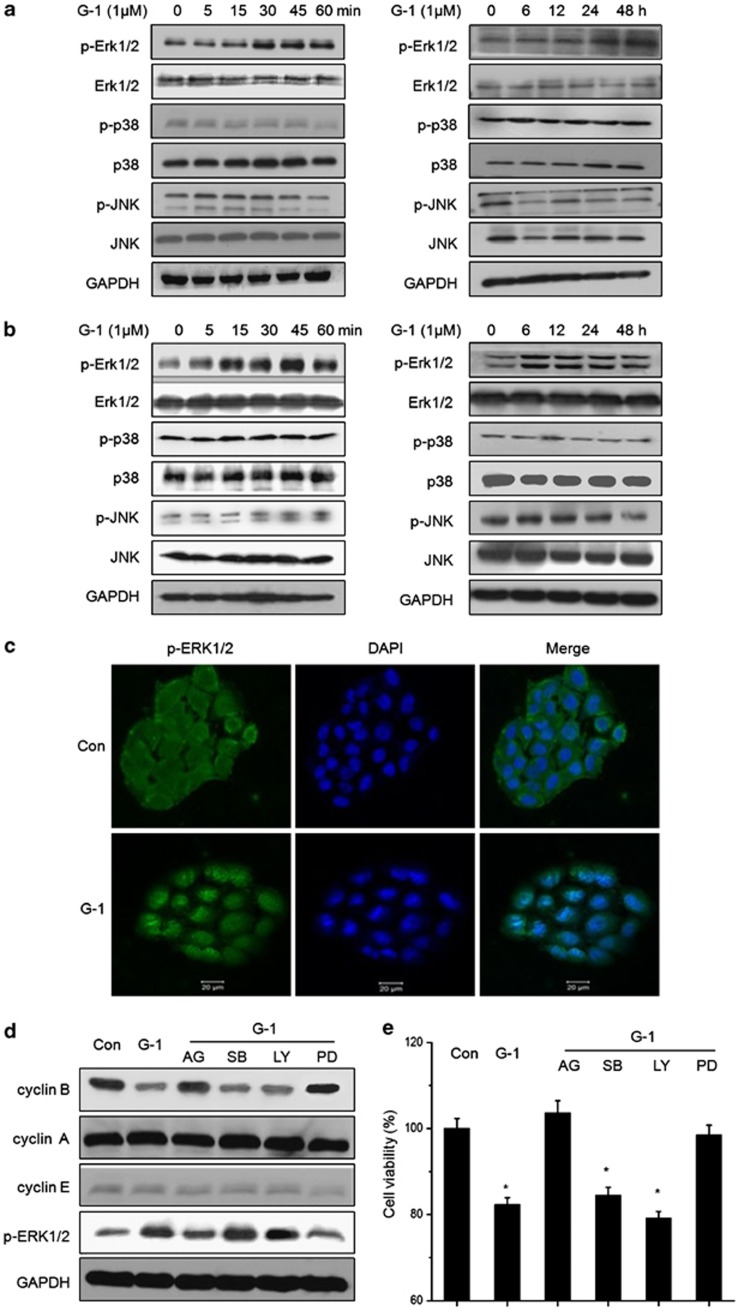
Activation of ERK by GPR30/EGFR mediated the growth arrest effects of G-1. SkBr3 (**a**) and MDA-MB-231 (**b**) cells were treated with 1 *μ*M G-1 for the indicated time periods, and then the phosphorylation and total protein levels of ERK1/2, JNK, and p-38 were detected by western blotting. (**c**) SkBr3 cells were treated with or without G-1 (1 *μ*M) for 24 h. After fixation, the cellular location of p-ERK1/2 (green) was examined by immunofluorescence staining and nuclei were stained with DAPI (blue). SkBr3 cells treated with 10 *μ*M MEK inhibitor PD98059 (PD), PI3K inhibitor LY294002 (LY), p38 MAPK inhibitor SB203580 (SB), or EGFR inhibitor AG1478 (AG) for 24 h, and then treated with 1 *μ*M G-1 for further 48 h, the protein levels of cyclin B, cyclin A, cyclin E, p-ERK1/2, and p21 were detected by western blotting (**d**), and the cell proliferation was measured by CCK-8 kit (**e**)

Recent studies indicated that the activation of GPR30 can inhibit the proliferation of cancer cells *via* MAPK signals.^10,16^ We assayed the effects of GPR30 agonist G1 on phosphorylation of MAPK in ER− breast cancer cells. As shown in Figure 5a, G-1 treatment can obviously activate ERK1/2 after treatment for 30 min. The sustained phosphorylation of ERK1/2 induced by G-1 lasted for more than 48 h, while the total levels of ERK1/2 protein were not changed. Such induced phosphorylation was also found in G-1-treated MDA-MB-231 cells (Figure 5b). JNK and p38 were observed at the same time. The levels of phosphorylated and total protein of JNK did not apparently change in the presence of G-1. While G-1 treatment slightly upregulated the levels of p-p38 in both SkBr3 and MDA-MB-231 cells (such as 6 h in SkBr3 and 12 h in MDA-MB-231). Further, we observed that 1 *μ*M G-1 treatment for 24 h significantly increased the nuclear localization of phosphorylated ERK1/2 in SkBr3 cells (Figure 5c). Our results suggested that activation of GPR30 by G-1 can induce a sustained phosphorylation of ERK1/2 and promote its nuclear translocation.

We next asked the downstream mediators responsible for cyclin B downregulation induced by G-1. SkBr3 cells were treated with MEK inhibitor PD98059, PI3K inhibitor LY294002, p38 MAPK inhibitor SB203580, or EGFR inhibitor AG1478, due to that activation of GPR30 can activate EGFR or PI3K/Akt signals^25^ and modulate the phosphorylation of ERK and p38 MAPK as revealed above. As shown in Figure 5d, both AG1478 and PD98059 significantly attenuated the G-1-induced downregulation of cyclin B, suggesting that inhibition of cyclin B by G-1 was primarily mediated by GPER/EGFR/ERK1/2. We also found that both AG1478 and PD98059 significantly attenuated the G-1-induced growth arrest of SkBr3 cells (Figure 5e), suggesting that inhibition effects of G-1 on ER− breast cancer cell proliferation are mediated by EGFR and sustained activation of ERK1/2. Taking together, these data suggested that sustained activation of ERK by GPR30/EGFR mediated the growth arrest effects of G-1.

### Upregulation of p21 by cross-talk of GPR30/EGFR and p53 was involved in G-1-induced ER− breast cancer cell growth arrest

**Figure 6:**
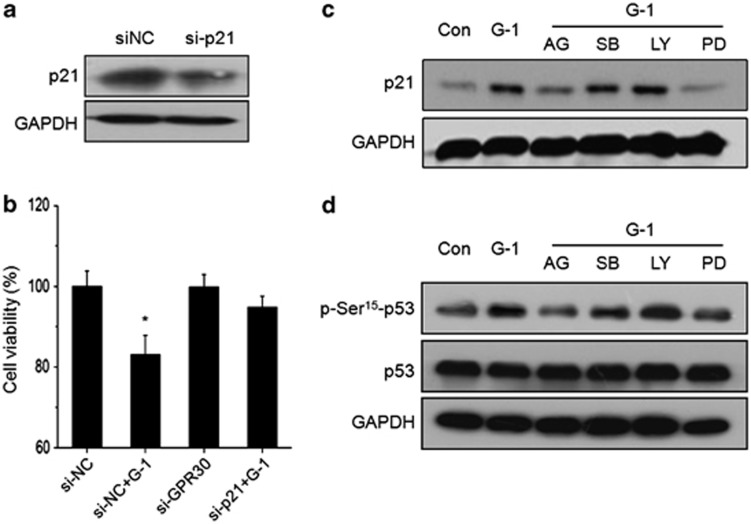
Upregulation of p21 by the cross-talk of GPR30/EGFR and p53 was involved in G-1-induced ER− breast cancer cell growth arrest. SkBr3 cells transfected with si-p21 or si-NC for 24 h, and then protein expression of p-21 was measured by western blotting (**a**). The transfected cells were then stimulated with or without G-1 (1 *μ*M) for another 24 h, the cell viability was assessed by CCK-8 kit (**b**). SkBr3 cells treated with 10 *μ*M MEK inhibitor PD98059 (PD), PI3K inhibitor LY294002 (LY), p38 MAPK inhibitor SB203580 (SB), or EGFR inhibitor AG1478 (AG) for 24 h, and then treated with 1 *μ*M G-1 for further 24 h (**c**) or 12 h (**d**), the protein levels of p21, p-Ser^15^-p53, and p53 were detected by western blotting

Above results revealed that activation of GPR30 by G-1 can upregulate expression of both p53 and p21^CIP1/WAF1^. To further investigate the roles of p21 in G-1-induced ER- breast cancer cell growth arrest, the expression of p21 was successfully silenced by si-p21 (Figure 6a). Silencing of p21 significantly attenuated the inhibition effects of cell proliferation in SkBr3 cells (Figure 6b).

The upregulation of p21 in ER− breast cancer cells can occur *via* p53, ERK1/2, and PI3K/Akt-dependent pathways.^26, 27, 28^ Then, we investigated the p53-independent upstream signals for G-1-induced upregulation of p21. We found that both AG1478 and PD98059, rather than LY294002 and SB203580, markedly attenuated the G-1-induced upregulation of p21, suggesting that G-1 can upregulate p21 expression *via* EGFR/ERK1/2 but not PI3K/Akt or p38-MAPK. It has been reported that both ERKs and p38-MAPK have a direct role in phosphorylation of p53 at serine 15 both *in vitro* and *in vivo*.^29^ Our results showed that both AG1478 and PD98059 obviously attenuated phosphorylation of p53 at Serine 15 in response to the G-1 treatment, while the protein expression of p53 was not affected. These data revealed that p21 upregulated by p53, EGFR/ERK1/2, and their cross-talk was involved in G-1-induced growth arrest of ER− breast cancer cells.

### The activation of GPR30 suppressed ER− breast cancer growth *in vivo*

**Figure 7:**
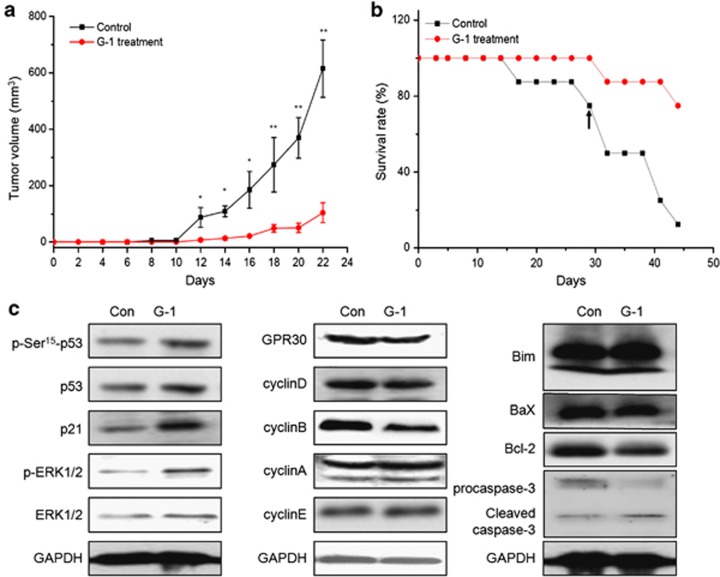
Activation of GPR30 by G-1 inhibited the ER− breast tumor xenograft growth *in vivo*. (**a**) After treatment with or without 1 *μ*M G-1 for 24 h, MDA-MB-231 cells injection subcutaneously into the fourth right mammary fat pad at the base of the nipple of nude mice with 50% Matrigel. Tumor size was measured at the indicated time intervals. (**b**) The survival rate of mice treated during the experiment. The survival rate of mice in G-1 treated group was significantly (*P*<0.05) higher than that of the control group from 27 days on after tumor implantation (indicated by the black arrow). (**c**) The proteins related to the growth inhibition effects of G-1 were determined by western blotting analysis in the tumor lysates from the control and G-1 treated group. *P<0.05 compared with control; **P<0.01 compared with control

To evaluate the role of GPR30 activation in tumor proliferation *in vivo*, we examined the ability of G-1 to suppress the growth of MDA-MB-231 tumor xenografts in nude mice. After injected for 10 days, control cells began to form measurable tumors, the volume of which increased with time (Figure 7a). In contrast, G-1 treated cells produced measurable tumors only after 18 days, after which they continuously grew. After 24 days, all mice were killed due to the large volume of tumor in the control group. During all the processes of experiments, the average size of tumor in G-1 groups was significantly (*P*<0.05) less than that of control ones (Figure 7a). Further, the reduction of tumor growth in G-1 group was associated with an increase in survival rate (Figure 7b). The survival rate of mice in G-1 group was significantly (*P*<0.05) greater than that of control mice from 27 days on after tumor implantation. Western blot analysis showed that G-1 treatment significantly enhanced the expression of p53, p21, ERK1/2, and cleaved caspase-3 while decreased the expression of cyclin B and Bcl-2 in tumors of the G-1 treated group (Figure 7c). In addition, we observed that ERK1/2 and p53 at Ser 15 was constitutively phosphorylated. These data suggested that activation of GPR30 inhibited the growth of ER− breast cancer growth in nude mice bearing MDA-MB-231 xenografts *via* proliferation suppression and apoptosis induction in tumor tissues.

## Discussion

**Figure 8:**
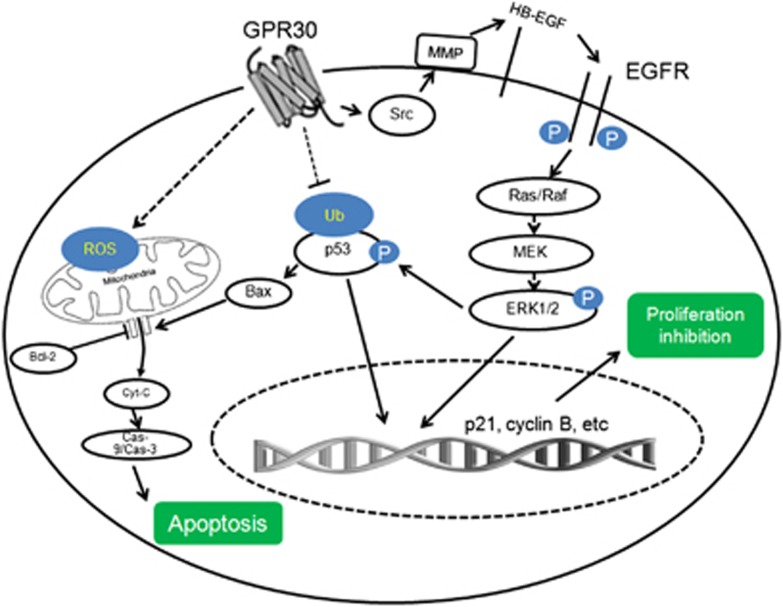
A proposed model to illustrate the mechanism of GPR30 mediated growth arrest and apoptosis of ER− breast cancer cells

Our present results revealed that activation of GPR30 can significantly inhibit ER− breast cancer cell proliferation by G2/M-phase arrest and mitochondrial-related apoptosis *via* multiple intracellular signaling pathways as summarized in Figure 8. In cultured cells, G-1 treatment decreased the expression of cyclin B, induced G2/M cell-cycle arrest, and caused mitochondrial-related apoptosis. Multiple signal pathways, such as upregulation of p53 *via* transcriptional and post-translational modifications, sustained activation of ERK1/2 *via* GPR30/EGFR signals, and upregulated p21 by p53, ERK1/2, and their cross-talks mediated the *in vitro* inhibition effects of G-1 on proliferation of ER− breast cancer cells. In MDA-MB-231 tumor xenografts in nude mice, initial single G-1 exposure can significantly delay *in vivo* growth and increase the survival rate of ER− breast cancer cells *via* proliferation suppression and apoptosis induction in tumor tissues.

Our results that activation of GPR30 inhibited the proliferation of ER− breast cancer cell is convenient with the growth arrest effects of GPR30 activation reported in many cancer types such as prostate,^16^ Leydig,^30^ urothelial cell,^31^ ovarian,^9^ and ER*α*-positive breast cancer.^10^ In contrast, there were also studies showed that activation of GPR30 is able to stimulate cell growth of endometrial,^11^ ovarian,^14^ and breast cancer cells.^32^ It should be noted that the stimulatory effects of GPR30 are stimulated with non-specific agonists such as estrogen and tamoxifen. This might be the possible reason for the controversial results observed. Further, cancer cells in which all three ERs (ER*α*, ER*β*, and GPR30) are expressed, it appears that the major proliferative effects are exerted and promoted by ER*α*,^10^ while activation of ER*β* and GPR30 is linked to growth arrest and apoptosis.^16^

The present study revealed that G-1 can induced G2/M cell-cycle arrest and mitochondrial-related apoptosis *via* activation of GPR30 in ER− breast cancer cells. The G-1 caused G2/M cell-cycle arrest was confirmed by the downregulation of cyclin B *via* a time-dependent manner. Two G2-checkpoint proteins cyclin B1 and Cdc2 have been reported to be reduced by G-1 treatment in prostate^16^ and breast^19^ cancer cells. The G2/M cell-cycle arrest induced by activation of GPR30 was also observed in ovarian^33^ and breast^34^ cancer cells. The G2/M cell-cycle arrest will lead to apoptosis *via* the intrinsic mitochondrial pathway.^35^ Our data confirmed that GPR30 activation can initiate the intrinsic apoptotic mechanism *via* downregulation of ΔΨm and upregulation of ROS. Bcl-2 exerts anti-apoptotic activities.^36^ Bax allows cytochrome c translocation to cytosol, activates procaspase 9, and then activates the executioner caspase 3.^37^ Three Bim isoforms, Bim_EL_, Bim_L_, and Bim_S_, all induce apoptosis, and the smallest variant Bim_S_ is the most potent inducer of apoptosis.^38,39^ All these events such as downregulation of Bcl-2, upregulation of Bax, Bim_S_, and cleaved caspase 3 were observed in ER− breast cancer cells in response to GPR30 activation, further confirmed that dysfunctions of mitochondria and mitochondrial-mediated pathways are involved in the inhibitory effects of G-1.

Our results revealed for the first time that the upregulation, stabilization, and nuclear translocation of p53 by activation of GPR30 is involved in G-1-induced growth arrest of ER− breast cancer cells. Although p53 is mutated (mtp53) in both MDA-MB-231 and SkBr3 cells, studies revealed that mtp53 can possess pro-oncogenic potential through coaggregation with other tumor suppressors and gain of functions such as transcriptional repression and activation.^40^ Targeting mtp53 has been reported to inhibit the proliferation of ER− breast cancer cells.^41^ In normal, p53 levels are low due to continuous MDM2-mediated ubiquitination and degradation.^42^ Our results indicated that G-1 treatment significantly downregulated the mRNA of MDM2 and upregulated the mRNA and protein of p53 *via* a time-dependent manner. Further, phosphorylation of p53 at Ser,^15^ which can prevent p53 from binding to its negative regulator MDM2^43^ and stimulate p53-dependent transactivation,^44^ was significantly increased by G-1 treatment. The protein stability of p53 is mainly regulated *via* ubiquitin mediated proteasomal degradation processes.^23^ We also found that G-1 treatment inhibited the p53 ubiquitination incrementally over time, whether it is attributed to the G-1 mediated downregulation of MDM2 and then decreased MDM2-mediated p53 ubiquitination still needs further study.^45^ Translocation from the cytoplasm to the nucleus, the key factor of transcription activities of p53, was also significantly inhibited by G-1 treatment. Finally, the knockdown assays confirmed that p53, which can be modified by G-1 *via* transcriptional and post-transcriptional pathways, mediated growth arrest effects of GPR30 activation on ER− breast cancer cells.

The rapid and sustained activation of ERK1/2 and its nuclear translocation stimulated by GPR30/EGFR signals mediated the growth arrest effects of G-1 on ER− breast cancer cells. We found that G-1 induced a rapid (since 15–30 min) and sustained (lasted for more than 48 h) phosphorylation of ERK1/2 in both SkBr3 and MDA-MB-231 cells. G-1 induced sustained ERK1/2 activation has also been observed in prostate,^16^ breast,^46^ and tumor Leydig cells.^30^ Phosphorylated ERK1/2 can translocate from the cytoplasm to the nucleus, which is observed in SkBr3 cells treated with G-1 (Figure 5c). It has been reported that sustained activation with nuclear accumulation of activated ERK1/2 transmitting antiproliferative signals.^47^ Activation of the MAP kinase ERK1/2 by GPR30 was mediated *via* EGFR transactivation.^25^ The present study revealed that GPR30/EGFR/ERK1/2 signals mediated G-1-induced downregulation of cyclin B and growth arrest of ER− breast cancer cells. The role of phosphorylation of p38 MAPK, which was slightly upregulated in SkBr3 and MDA-231 cells, was not involved in G-1-induced growth arrest in the present study and needed further study.

The upregulation of p21 *via* p53, EGFR/ERK1/2, and their cross-talk was suggested to be involved in G-1-induced growth arrest of ER− breast cancer cells. Recent studies revealed that upregulation of p21 was involved in G2/M cell-cycle arrest^48^ and associated with nuclear translocation of G2-checkpoint regulators.^49^ The p21-induced downregulation of cyclin B has been linked to G2/M arrest.^50,51^ We found that G-1 treatment significantly increased the expression of p21 *via* a time-dependent manner, further, siRNA knockdown of p21 effectively blocked the G-1-induced inhibition of cell growth. This was confirmed by the study that p21-mediated the G-1-induced cell-cycle arrest at the G2 phase in PC-3 cells.^16^ Further, we found that GPR30/EGFR/ERK1/2 mediated the G-1-induced upregulation of p21. High intensity and sustained activation of ERK1/2 can induce high expression of p21.^52^ At the same time, GPR30/EGFR/ERK1/2 also mediated the phosphorylation of p53 at Ser 15 in ER− breast cancer cells, which might be induced by the direct interaction of ERK and p53 on Ser 15.^53^ Phosphorylation of p53 at Ser 15 has been shown to be involved in activating p53^43^ and activating the transcription of p21.^54^ Collectively, our data revealed that p53, EGFR/ERK1/2, and their cross-talk upregulated the expression of p21 and mediated the growth arrest effects. The inhibition effects on ER− breast cancer proliferation and mechanisms illustrated *in vitro* were also confirmed by the *in vivo* study, in which initial single G-1 exposure markedly inhibited growth of ER− breast cancer cell xenograft, enhanced the expression of p53, p21, ERK1/2, and cleaved caspase-3 while decreased the expression of cyclin B and Bcl-2 in tumors.

In conclusion, our present study revealed that the activation of GPR30 can inhibit the proliferation *in vitro* and *in vivo* through the mechanisms summarized in Figure 8. Although further studies are needed, our results pointed out how GPR30 and its agonists such as G-1 can be considered as a potential new pharmacological tool to reduce the growth of ER− breast cancer. Considering that there is no efficiency therapy targets for ER− breast cancer, the present study not only strongly suggested that GPR30 can be considered as a potential important target but also provided G-1 as a drug candidate for ER− breast cancer therapy.

## Materials and Methods

### Reagents

PD 98059 (PD, MAPK/ERK kinase agonist) and AG 1478 (AG, EGFR antagonist) were purchased from Selleck Chemicals (Houston, TX, USA). G-1 (GPR30 agonist) and other chemicals were of reagent grade or better and purchased from Sigma Chemical Co. (St. Louis, MO, USA) unless otherwise noted. Monoclonal antibodies against cyclin A, cyclin B1, cyclin E, Bcl-2, ERK1/2, Caspase 3, Bim, p21, p53, and GAPDH were purchased from Cell Signaling Technology Inc. (Beverly, MA, USA). Antibodies against p-ERK1/2, and p-p53 (Ser 15) were purchased from Bioworld Technology, Inc. (Minneapolis, MN, USA). Horseradish peroxidase-conjugated secondary antibody from Santa Cruz Biotechnology (Santa Cruz, CA, USA).

### Cell lines and culture

ER− breast cancer cell lines MDA-MB-231 and SkBr3 purchased from the American Type Culture Collection (Manassas, VA, USA) were cultured in RPMI medium 1640 (Invitrogen Corporation, Carlsbad, CA, USA) supplemented with 10% heat-inactivated fetal Bovin serum, 100 U/ml penicillin, and 10 *μ*g/ml streptomycin at 37°C in a 5% CO_2_ atmosphere. Medium was replaced with phenol red-free medium 24 h before experiments to remove the estrogen-like activity of phenol red.

### Cell viability assays

Viability of cells was evaluated by use of the CCK-8 kit (Dojindo Molecular Technologies, Gaithersburg, MD, USA) according to the previously described procedures.^55,56^

### Assays of cell cycle and apoptosis

Cells were plated at a density of 1 × 10^6^ per well on six-well plates and then synchronized at the G1/S transition by a double TdR block, as follows: 16 h block with 2.5 *μ*M TdR (Sigma), 10 h release followed by the second block for 16 h. Then cells were treated with G-1 for indicated times, collected into flow cytometry tubes, washed with PBS, fixed with 70% ethanol overnight at 4°C, incubated with propidium iodide (50 *μ*g/ml), and analyzed by a Coulter Epics XL Flow Cytometry System (Beckman-Coulter, Miami, FL, USA). For cell apoptosis analysis, after treatment with G-1, both the suspension and the adherent cells were collected, stained with Annexin V-FITC for 15 min and propidium iodide for 5 min, and analyzed immediately by flow cytometry using FL1 (Em: 525 nm) and FL3 (Em: 670 nm).

### Determination of ΔΨm and ROS

JC-1 probe was employed to measure mitochondrial depolarization in ER− breast cancer cells. Cells were treated with G-1 for 24 h. JC-1 staining solution (5 *μ*g/ml) was added at 37°C for 20 min. After washing with PBS twice, mitochondrial membrane potentials were monitored by determining the relative amounts of dual emission from a multiple fluorescence reader. The fluorescence in cells was quantitatively analyzed by FCM. Mitochondrial depolarization is depicted by an increase in the green/red fluorescence intensity ratio. ROS were monitored with the oxidation-sensitive fluorescent probe 2′,7′-dichlorodihydrofluorescein diacetate (DCF-DA).

### Western blotting and immunoprecipitation analysis

Western blotting was performed as previously described.^57^ For immunoprecipitation, cellular lysate (500 *μ*g) was used to immunoprecipitate, and western blotting was conducted to examine ubiquitination or interaction of proteins. GAPDH (10% input) was used as an input control.

### Quantitative real-time PCR

After treatment as indicated, total mRNA of cells was extracted with TRIZOL reagent. First strand of cDNA was generated from 2 *μ*g total RNA using oligo-dT primer and Superscript II Reverse Transcriptase (GIBCO BRL, Grand Island, NY, USA). Quantitative real-Time PCR was run on an iCycler (Bio-Rad, Hercules, CA, USA) using validated primers and SYBR Premix Ex Taq II (Takara, Japan) for detection. The cycle number when the fluorescence first reached a preset threshold (Ct) was used to quantify the initial concentration of individual templates for expression of mRNA of genes of interest. Transcripts of the housekeeping gene GAPDH in the same incubations were used for internal normalization. Primer pairs were as follows: cyclin A, forward 5′-TGG ACC TTC ACC AGA CCT AC-3′ and reverse 5′-GGT TGA GGA GAG AAA CAC CA-3′ cyclin B, forward 5′-CCA GAG GTG GAA CTG GAT G-3′ and reverse 5′-GGG CTT GGA GAG GGA GTA TC-3′ cyclin D, forward 5′-GAG GAA GAG GAG GAG GAG GA-3′ and reverse 5′-GAG ATG GAA GGG GGA AAG AG-3′ cyclin E, forward 5′-GCA GTA TCC CCA GCA AAT C-3′ and reverse 5′-TCA AGG CAG TCA ACA TCC A-3′ p53, forward 5′-GGT GGT GCC CTA TGA GCC G-3′ and reverse 5′-TCC TCT GTG CGC CGG TCT C-3′ MDM2, forward 5′-GGC ATG CTT CAC ATG TGC AA-3′ and reverse 5′-GTA CAA TCA TTT GAA TTG GTT GCC-3′ GAPDH, forward 5′-GCA CCG TCA AGG CTG AGA AC-3′ and reverse 5′-TGG TGA AGA CGC CAG TGG A-3′.

### Immunofluorescence

Fifty percent confluent cells were cultured on confocal dishes and then exposed to G-1 for the indicated time. Then, cells were washed three times with PBS, fixed in 4% paraformaldehyde for 20 min, and permeabilized with 0.3% Triton X-100 for 10 min. After blocking with goat serum for 2 h, cells were incubated for 1 h with antibody against phosphorylated ERK1/2 or p53, respectively. Then, dishes were washed three times with PBS and incubated with Alexa Fluor 488 or Alexa Fluor 594-conjugated secondary antibodies (1:1000 dilution) for 1 h at room temperature. Nuclei were stained with DAPI (10 mg/ml) for 10 min. Samples were examined with Confocal Laser Scanning Microscopy (Zeiss, Jena, Germany) to analyze expression and nuclear translocation of ERK1/2 and p53.

### RNA interference

Cells were seeded on a 6-well plate (2 × 10^5^ cells/well) and left in culture until the next day. They were then transfected with 100 pmol siRNA oligomer mixed with lipofectamine 2000 reagent in serum reduced medium according to the manufacturer's instructions. Medium was changed to complete culture medium 6 h later, and the cells were incubated at 37°C in a CO_2_ incubator for another 24–48 h before harvest.

### Tumorigenesis assay

Nude mice were purchased from the Sun Yat-sen University (Guangzhou, China) Animal Center and raised under pathogen-free conditions. All animal studies were conducted in accordance with institutional guidelines for the care and use of experimental animals. 2 × 10^6^ MDA-MB-231 cells were treated with G-1(1 *μ*M) for 24 h before injection subcutaneously into the fourth right mammary fat pad at the base of the nipple of nude mice (*n*=8) with 50% Matrigel (BD Bioscience, Bedford, MA, USA). Untreated cells were injected contralaterally. Tumor growth and body weight were monitored every 2 days. The tumor volume was calculated using the formula *V*=1/2 × larger diameter × (smaller diameter)^2^. At the end of treatment, the animals were killed, and the tumors were removed and weighed for use in histology and western blotting analysis.

### Statistical analysis

All values were reported as mean±S.D. of three independent experiments unless otherwise specified. Data were analyzed by two-tailed unpaired Student's *t*-test between two groups and by one-way ANOVA followed by Bonferroni test for multiple comparison involved. The statistical analyses were performed using SPSS 17.0 (SPSS, Inc, Chicago, IL, USA) for Windows. A *P*-value of <0.05 was considered to be statistically significant.

## Abbreviations

ER: estrogen receptor
EGFR: epidermal growth factor receptor
GPR30: G protein-coupled receptor 30
MAPK: mitogen-activated protein kinase
ROS: reactive oxygen species
PI3K: phosphoinositide 3-kinase
Δ Ψm: mitochondrial membrane potential
