# Correction: The Activation of G Protein-Coupled Receptor 30 (GPR30) Inhibits Proliferation of Estrogen Receptor Negative Breast Cancer Cells in vitro and in vivo

**DOI:** 10.1038/s41419-025-07995-1

**Published:** 2025-10-21

**Authors:** W. Wei, Z-J Chen, K-S Zhang, X-L Yang, Y-M Wu, X-H Chen, H-B Huang, H-L Liu, S-H Cai, J. Du, H-S Wang

**Affiliations:** 1 Guangdong Provincial Key Laboratory of New Drug Design and Evaluation, Department of Microbial and Biochemical Pharmacy, School of Pharmaceutical Sciences, Sun Yat-sen University, Guangzhou, 510006 China; 2 Department of Pharmacy, Sun Yat-sen University Cancer Center; State Key Laboratory of Oncology in South China; Collaborative Innovation Center for Cancer Medicine, Guangzhou, 510060 China; 3 Department of Pharmacy, Sun Yat-sen Memorial Hospital, Sun Yat-sen University, 107 Yanjiang West Road, Guangzhou, 510120 China; 4 Key Laboratory of Tropical Disease Control (Ministry of Education), Guangdong Institute of Gastroenterology and the Sixth Affiliated Hospital, Institute of Human Virology, Sun Yat-sen University, Guangzhou, 510655 China; 5 Department of Pharmacology, School of Pharmaceutical Sciences, Jinan University, Guangzhou, 510632 China

Correction to: *Cell Death and Disease* 10.1038/cddis.2014.398, published online 2 October 2014

Our internal review identified one duplication within our manuscript, specifically where the p-p38 band in Figure 5a overlaps with the cyclin E band in Figure 5d. This issue likely arose during the figure assembly process due to a copy-paste error, particularly when using the template of Figure 5d to prepare Figure 5a, resulting in the cyclin E band from Figure 5d overlaying the p-p38 band in Figure 5a. We have thoroughly reviewed our original data. However, due to the significant time elapsed since the experiment and the graduation of the involved students, we were able to locate the original data for the cyclin E band in Figure 5d, but not the original p-p38 band data for Figure 5a.

To rigorously address this, we repeated the experiments related to Figure 5a three times. Antibodies used: p-p38 (Santa Cruz, sc-166182), p38 (Santa Cruz, sc-81621), GAPDH (CST, 5174S). Experiments were repeated under identical conditions as the original study (SkBr3 cells were treated with 1 μM G-1 for the indicated time periods, and then the phosphorylation and total protein levels of p-38 were detected by Western-blotting).”

The new results (see attached raw data) consistently demonstrate that G-1 does not affect p-p38 levels, which aligns with the original conclusion. Nonetheless, to uphold the highest standards of data accuracy, we propose the following correction:

- **Replace Figure 5a** with representative data from our replicated experiments, clearly labeled as such in the corrected figure.

Due to an oversight in figure selection, the original representative image of Figure 5 A (p-p38) is incorrect. However, this does not affect the experimental conclusions. Therefore, it is necessary to correct Figure 5 A (p-p38).

Figure 5 A
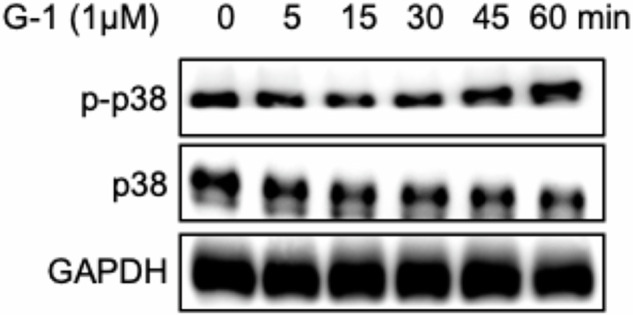


**Figure 5 A** SkBr3 cells were treated with 1 μM G-1 for the indicated time periods, and then the phosphorylation and total protein levels of p-38 were detected by Western-blotting.

## Supplementary information

Supporting data for correction

