# Supplementary material for: Correction: The Activation of G Protein-Coupled Receptor 30 (GPR30) Inhibits Proliferation of Estrogen Receptor Negative Breast Cancer Cells in vitro and in vivo
Source: Cell Death Dis. 2025 Oct 21;16(1):739. doi: 10.1038/s41419-025-07995-1 (PMC12540899; doi:10.1038/s41419-025-07995-1)
Supplement: Supplementary file 1 — Supporting data for correction [file 41419_2025_7995_MOESM1_ESM.pptx]

## Slide 1
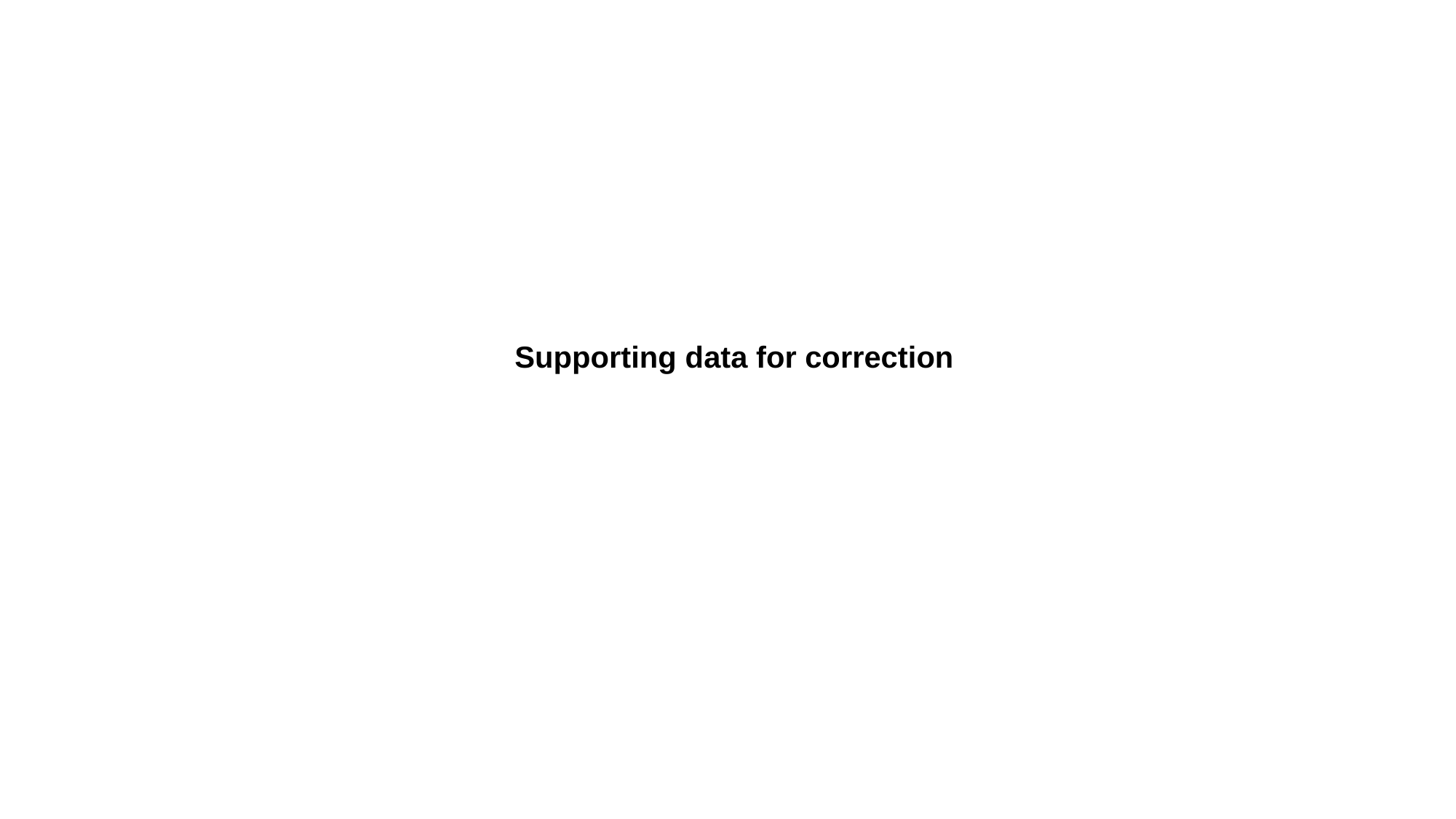

Supporting data for correction

## Slide 2
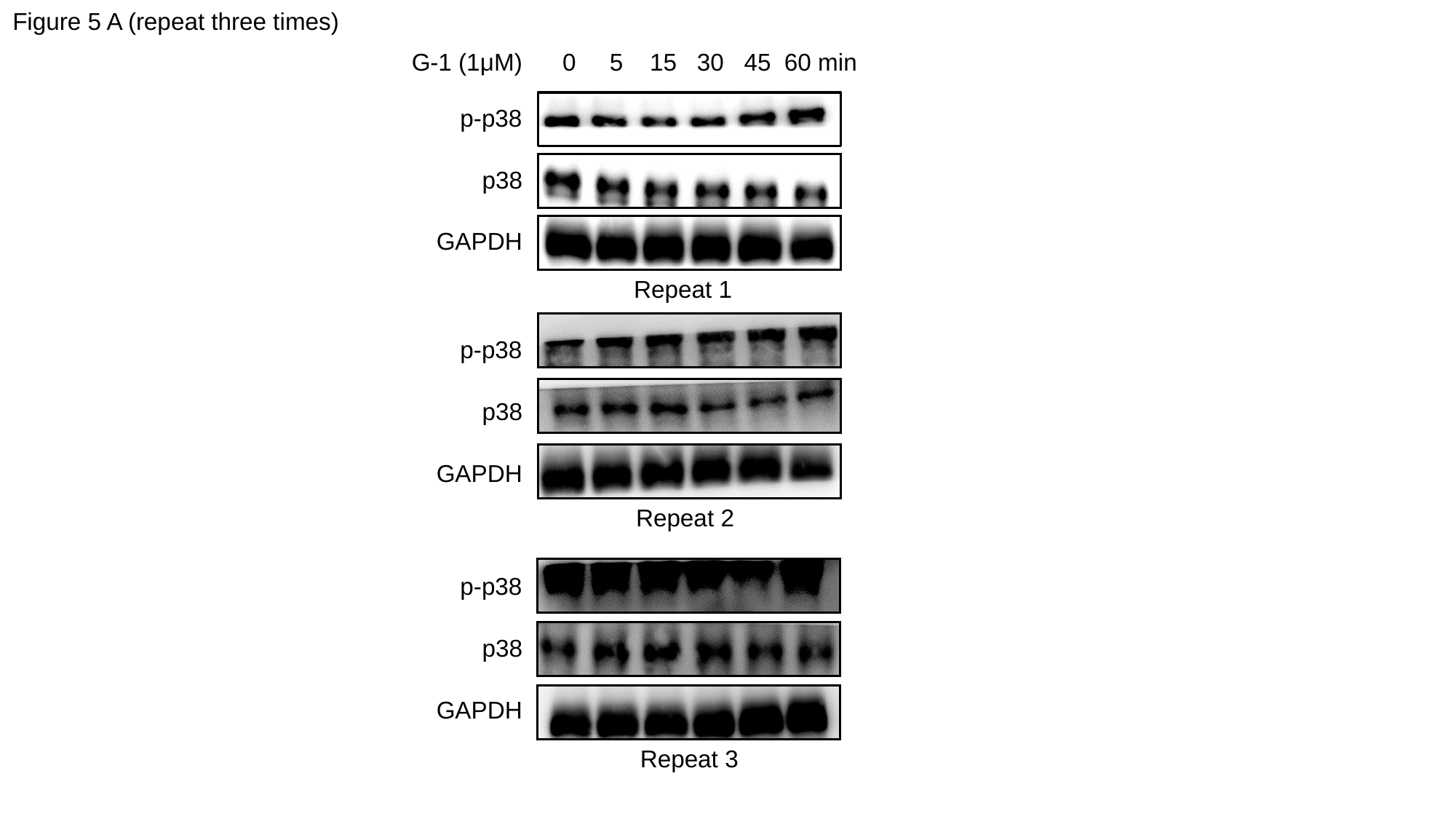

Figure 5 A (repeat three times)
G-1 (1μM) 0 5 15 30 45 60 min
p-p38
p38
GAPDH
Repeat 1
p-p38
p38
GAPDH
Repeat 2
p-p38
p38
GAPDH
Repeat 3

## Slide 3
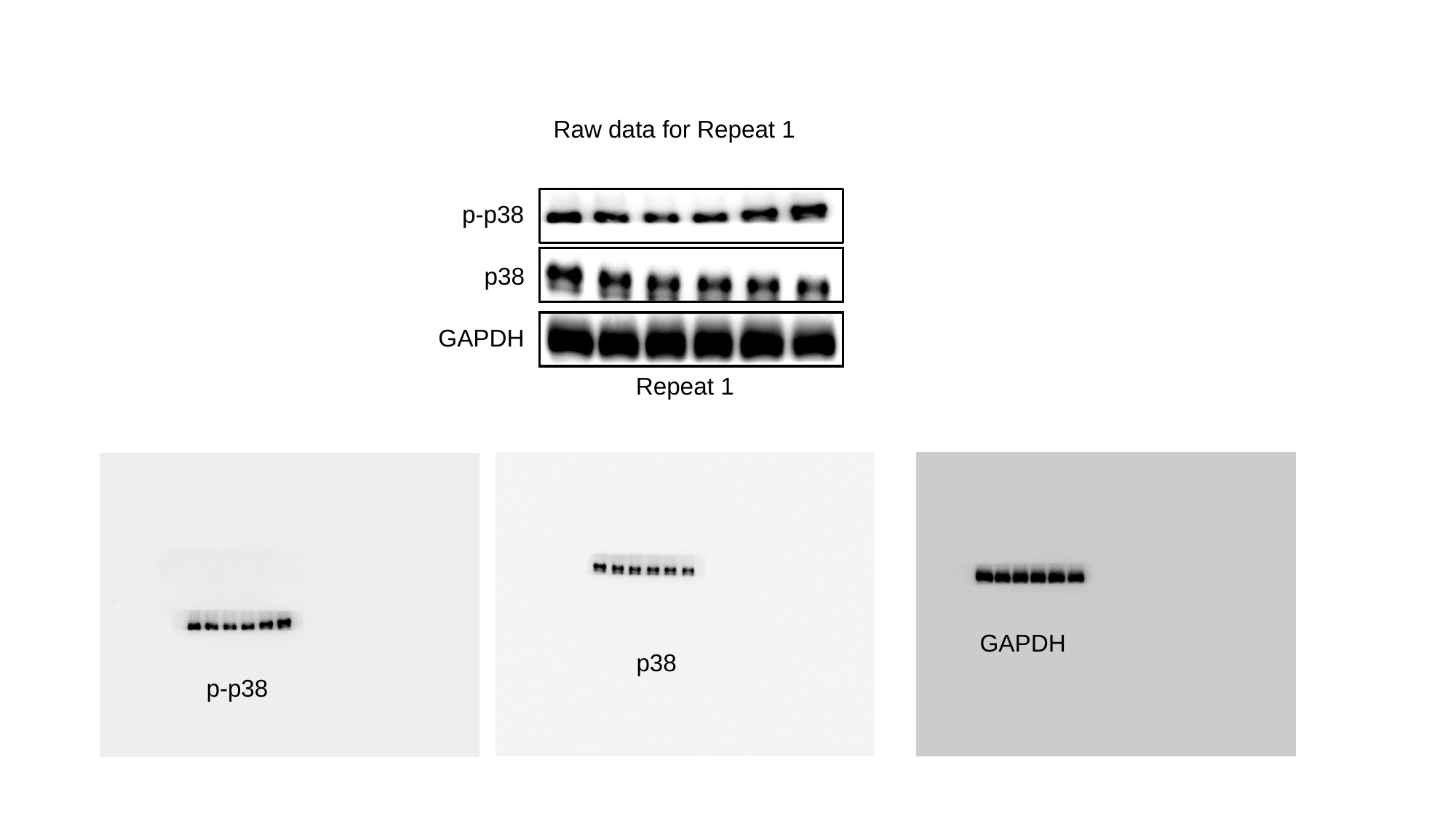

Raw data for Repeat 1
p-p38
p38
GAPDH
Repeat 1
GAPDH
p38
p-p38

## Slide 4
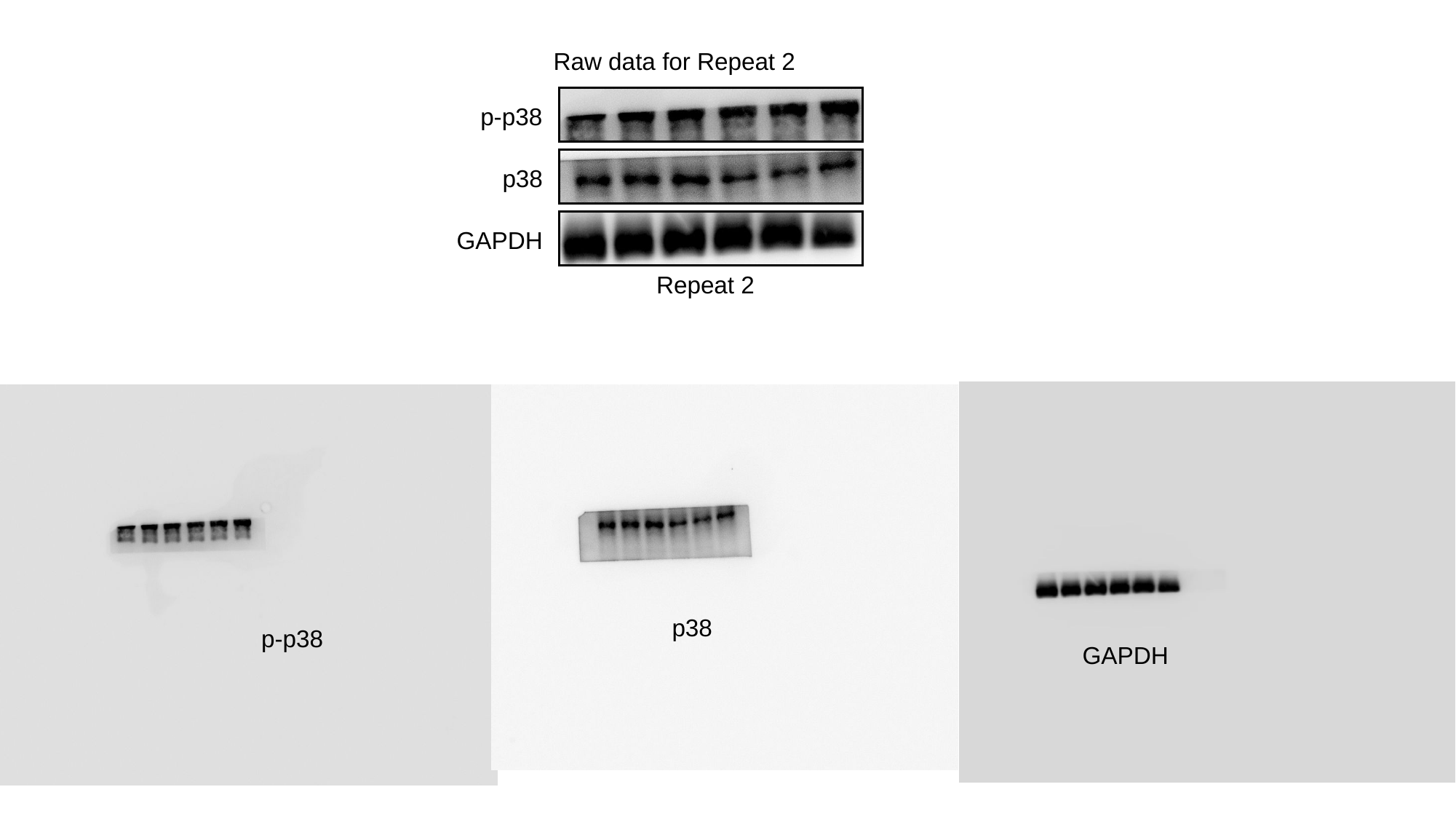

Raw data for Repeat 2
p-p38
p38
GAPDH
Repeat 2
p38
p-p38
GAPDH

## Slide 5
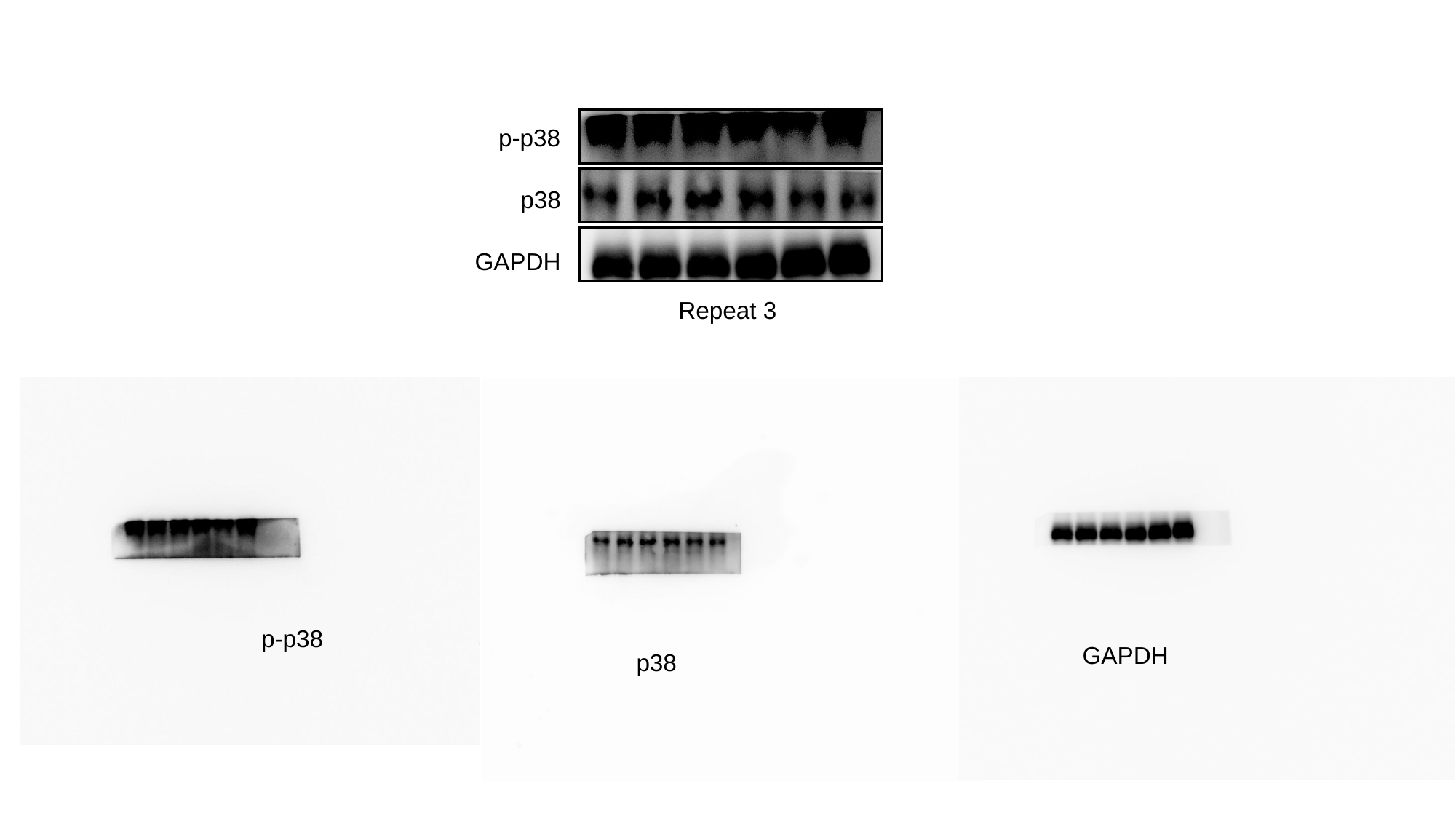

p-p38
p38
GAPDH
Repeat 3
p-p38
GAPDH
p38
